# A multi-perspective study of Perceived Inclusive Education for students with Neurodevelopmental Disorders

**DOI:** 10.1007/s10803-022-05643-7

**Published:** 2022-07-04

**Authors:** Emma Leifler, Anna Borg, Sven Bölte

**Affiliations:** 1 Karolinska Institutet Center of Neurodevelopmental Disorders (KIND), Centre for Psychiatry Research, Department of Women’s and Children’s Health, Karolinska Institutet, & Stockholm Health Care Services, Region Stockholm, Stockholm, Sweden; 2 Department of Pedagogical, Curricular and Professional Studies, University of Gothenburg, Göteborg, Sweden; 3 Child and Adolescent Psychiatry, Stockholm Health Care Services, Region Stockholm, Stockholm, Sweden; 4 Curtin Autism Research Group, School of Allied Health, Curtin University, Perth, Western Australia

**Keywords:** Inclusive education, Autism, ADHD, Consensus, Assessment, Teachers, Parents

## Abstract

Consensus is often a prerequisite for communities to develop initiatives to improve practice and create a future together. We investigated the consensus around the perceived educational inclusion of autistic and other neurodivergent students, their caregivers, and their teachers. Seventeen triads of informants plus two single students from mainstream secondary and high schools in Sweden underwent the standardized INCLUSIO interview operationalizing educational inclusion domains. Inclusive practice was reported across groups to be poorly to sufficiently developed for different domains and measures. Discrepancies were extensive between informants and most pronounced for students and parents versus teachers. The findings highlight limited consensus about inclusive education in practice and suggest enhanced participation of neurodivergent students and their parents to improve inclusive education implementation.

Inclusive education is a core component of human rights, fostering equal learning opportunities and social justice (Armstrong, 2005). International declarations agree that inclusive education should entail the accommodation of all students’ needs, no matter the prerequisites, and avoid stigma (European Agency for Special Needs and Inclusive Education, 2018; UN, 2016; UNESCO, 1994, 2005). Many high- and middle-income countries have introduced inclusive educational policies embracing these statements, aiming to reduce segregated education of divergent students (Hardy & Woodcock, 2015). The coming into effect of these policies has been paralleled by an increasing number of children diagnosed with autism, attention-deficit hyperactivity disorder (ADHD), and other neurodevelopmental disorders (NDDs) (Danielson et al., 2016; Maenner et al., 2021). Thus, considerably more students diagnosed with NDDs currently attend mainstream schools (Florian, 2014). Guidance for school professionals regarding how to implement inclusive education and the provision of resources to facilitate inclusion vary between countries and schools and are often limited (Gitschthaler et al., 2021; Schwab, 2020). Furthermore, dissent exists about what defines inclusive education, how it should be translated into practice, what level of inclusion is meaningful, and what has been accomplished (Nilholm, 2021; Nilholm & Göransson, 2017).

Despite increasing evidence for and against certain approaches to facilitating the educational inclusion of students with NDDs (Crosland & Dunlap, 2012; Leifler et al., 2022; Lovett & Nelson, 2021), continuous challenges to achieve educational inclusion have been reported, particularly for students with NDDs (Pellicano et al., 2018). In a previous study, we examined inclusive practices for students with NDDs in Sweden, as reported by a large sample of school staff across various professional backgrounds and school types (Bölte et al., 2021). Participants reported poorly or modestly implemented educational inclusion measures, particularly the absence of mentor systems and coordination with outside school services for implementing support. Importantly, there were considerable differences between the reports by principals, teachers, special educators, youth workers, and school health team members. Substantial variation in perceived inclusion by neurotypical and neurodivergent people and their families has also been found in other studies (Jones et al., 2022).

However, consensus is crucial for making decisions that are in the best interest of a community (Dressler, 2006). Hence, discovering and addressing incongruent views is key, as they are likely to cause miscommunication, false expectancies, or the blocking of decisions leading to fruitful community development. In addition, as the attitudes of some stakeholders could be implicitly or explicitly prioritized over those of others by decision makers, for example, those of professionals over those of individuals with NDDs and their caregivers, the needs of the target groups that policies claim to serve might be neglected. Therefore, this study’s objective was to investigate educational inclusion by assessing the implementation of inclusive measures from the perspective of students with NDDs, their caregivers, and their teachers. Based on our experience and previous findings, we hypothesized that limited consensus would exist among groups; specifically, the opinions of neurodivergent students and their parents about implemented inclusive measures would be less favorable than those of their teachers.

## Methods

### Design

This study was approved by the Swedish Ethical Review Authority. Informed consent was signed prior to participation. We conducted an ex-post facto quasi-experimental and correlational study. Inclusive educational practices, as reported by three groups (neurodivergent students, their parents, their teachers), were examined using standardized interview findings. Group differences and convergence of reported experienced educational inclusion were analyzed. Data collection started in the fall of 2019 and was finalized in the fall of 2021. Due to the social distancing regulations during periods of the COVID-19 pandemic, data collection was performed during both face-to-face meetings and in remote form using Zoom video conferencing, Google Meet, or telephone.

## Participants

The sample consisted of groups of neurodivergent students, their parents, and their teachers recruited in the form of 17 complete triads. Specifically, included parents and teachers were the caregivers and educators of the specific target students. In addition, two solitary students were included. Hence, the study had 53 participants: 19 students (4 female, 15 male), 17 of their parents (13 mothers, 4 fathers), and 17 of their teachers. The students’ ages ranged from 15 to 20 years (*M* = 17.0, *SD* = 1.6). The average professional experience among the teachers was 14.7 years (*SD* = 8.0, *range* = 2 to 32 years). The participants originated from seven mainstream secondary and high schools across an urban municipality on the west coast of Sweden. Participants were recruited via requests sent to the schools and forwarded to students, parents, and teachers by the principals. Three of the neurodivergent participants had a self- and parent-reported primary clinical diagnosis of autism spectrum disorder (ASD), seven reported a double diagnosis of ASD and ADHD, and nine reported an ADHD diagnosis. There were additional co-existing neurodevelopmental and psychiatric difficulties reported in the neurodivergent group: dyslexia (3), obsessive compulsive disorder (1), and post-traumatic stress disorder (1).

## Instrument

Participants completed a 21-item standardized interview version derived from the INCLUSIO scale (Bölte et al., 2021). The original INCLUSIO is a school staff report questionnaire operationalizing inclusive educational measures for neurodivergent students in mainstream schools. It operationalizes inclusive actions on eight domains (see Table 1). The content validity of the INCLUSIO scale was established based on a cross-professional Delphi process in a group of NDD, education, and inclusion professionals and experts. Reliability in terms of internal consistency (Cronbach’s alpha) for all 61 questionnaire items in a large sample of 4,778 school staff was α = 0.87, and between α = 0.70 to 0.89 for its domains (Bölte et al., 2021). For the current study, INCLUSIO was transformed to an interview format using a Delphi process to ensure a comparable comprehension of item meanings across students, teachers, and parents, and was reduced to a selection of 21 items (see Table 1) that would be possible to evaluate across informants. For the generated interview version, internal consistency in the given sample was α = 0.89. INCLUSIO items are Likert-scaled and scored from 0 to 3. Items can be answered with “yes” (score 3, indicating inclusive practice), “rather yes” (score 2, indicating moderate inclusive practice), “rather no” (score 1, indicating doubtful inclusive practice), “no” (score 0, indicating no inclusive practice), and “don’t know” (scored 9 [and converted to 0], indicating no knowledge of inclusive practice). Item scores are summed to domain scores and a total score.

### Data Analysis

Interview data collected from students, parents, and teachers were analyzed in SPSS/Win. 27. Descriptive statistics (*M*, *SD*) for all 21 INCLUSIO interview items, the 8 domains, and the total score are provided for the 3 groups. Inference statistics included three steps. First, groups were compared for reported inclusive practices, applying general linear modeling (MANOVA) across INCLUSIO total, domain, and item scores, followed by post hoc Tukey tests for single group comparisons. Then, Kappas were computed using crosstabs within the total sample across groups for all INCLUSIO items to determine the degree of agreement between groups for single inclusive measures. Finally, Pearson intercorrelations were computed between item and domain scores, on the one hand, and total scores, on the other, in the whole sample to investigate relations between single inclusive measures and domains and rated overall educational inclusion. An alpha level of 5% was adopted for all statistics, and one-tailed tests were conducted for directional hypotheses. Given the sample size and alpha, the power (1-beta) to detect differences and associations in this study was good to high (71 to 0.99) for large effects, low to good (0.33 to 0.73) for medium effects, and low (0.10 to 0.17) for small effects (G*Power).

**Table 1:** INCLUSIO Domains and Items: Comparisons Between Ratings by Students, Parents, and Teachers

	Students (S) M *(SD)*	Parents (P) M *(SD)*	Teachers (T) M *(SD)*	F	P	Eta^2^	Post-hoc
**Total score**	32.5 *(9.3)*	33.3 *(14.6)*	42.5 *(10.2)*	5.2	0.001	0.23	T > S = P
**Assessment of support needs** (max. 9)	4.8 *(1.7)*	5.5 *(2.6)*	5.8 *(2.5)*	2.2	0.49	0.11	
*Recommendations from clinical services are used for support planning*	1.8 *(1.2)*	1.1 *(1.2)*	1.8 *(0.7)*	1.9	0.078	0.07	
*There is a specific and accessible support plan document for the students*	1.5 *(0.9)*	2.2 *(1.2)*	2.1 *(1.2)*	2.6	0.043	0.09	P = T > S
*Staff involved in support plans meet regularly and support plans are evaluated*	2.0 *(1.1)*	2.1 *(1.2)*	1.8 *(1.1)*	0.3	0.371	0.01	
**Use of individualized support** (max. 12)	6.4 *(3.2)*	7.0 *(3.4)*	9.5 *(1.8)*	5.1	0.002	0.23	T > S = P
*Students are offered alternative options to demonstrate knowledge* ^a*^	1.9 *(0.9)*	1.9 *(0.8)*	2.8 *(0.4)*	9.0	0.0005	0.26	T > S = P
*School rules are adapted to students’ needs* ^b^	1.3 *(1.2)*	1.94 *(1.0)*	2.3 *(1.0)*	4.3	0.09	0.15	
*Students receive the individual special education support needed*	1.5 *(1.1)*	1.6 *(1.2)*	2.1 *(1.1)*	1.2	0.148	0.05	
*Everyday individual adaptations in the classroom and schedule are provided*	1.7 *(1.1)*	1.6 *(1.1)*	2.3 *(0.6)*	2.7	0.036	0.10	T > S = P
**Implementation of a structured learning environment** (max. 9)	4.1 *(2.0)*	4.1 *(2.6)*	4.8 *(2.4)*	1.4	0.127	0.07	
*The school uses visualization of schedules and time* ^c*^	2.2 *(1.0)*	1.2 *(1.1)*	1.7 *(1.1)*	3.6	0.017	0.12	S > P = T
*Students are offered organizational aid* ^d^	1.0 *(0.8)*	1.0 *(1.0)*	1.9 *(1.1)*	5.7	0.003	0.18	T > S = P
Changes to procedures are communicated to NDD* students as early as possible	0.9 *(1.0)*	1.8 *(1.0)*	1.1 *(1.1)*	3.8	0.014	0.13	P > S = T
**Individual changes applied to schedule/teaching** (max. 6)	3.1 *(1.9)*	2.2 *(1.5)*	3.6 *(1.4)*	3.5	0.011	0.17	T > P
*Students’ interests are integrated in teaching*	1.1 *(1.0)*	1.4 *(1.0)*	1.7 *(1.1)*	1.2	0.149	0.05	
*Strategies for handling stressful situations are provided*	2.0 *(1.2)*	0.8 *(0.7)*	1.9 *(1.0)*	7.4	0.001	0.23	S = T > P
**Functional response to behavioral characteristics** (max. 6)	3.3 *(1.9)*	3.2 *(1.6)*	4.6 *(1.6)*	4.2	0.005	0.19	T > P = S
The staff gets time to discuss NDD* students’ behavior and support plans	1.5 *(1.3)*	1.3 *(1.1)*	2.4 *(0.9)*	4.9	0.005	0.16	T > S = P
*The school offers space for rest and withdrawal*	1.8 *(1.1)*	1.9 *(1.0)*	2.2 *(1.1)*	0.5	0.315	0.02	
**Cooperation with parents (max. 9)**	6.1 *(2.0)*	5.3 *(2.8)*	6.4 *(2.3)*	2.2	0.495	0.11	
*There is a mutual exchange of knowledge about students with NDDs between home and * *school*	2.1 *(1.2)*	1.8 *(1.1)*	2.5 *(0.9)*	1.6	0.107	0.06	
*The school uses caregivers’ knowledge to optimize support*	1.5 *(1.1)*	1.8 *(1.2)*	2.1 *(1.0)*	1.1	0.173	0.04	
*There are regular exchanges between caregivers and responsible staff around students with NDDs*	2.4 *(0.8)*	1.8 *(1.2)*	1.9 *(1.0)*	2.1	0.067	0.08	
**Consideration of peer-relations (max. 6)**	2.0 *(1.5)*	3.2 *(2.5)*	4.1 *(1.2)*	5.2	0.001	0.23	T > S
*In group work, the composition of the group takes into account knowledge of students with NDDs*	1.3 *(0.9)*	1.8 *(1.4)*	2.5 *(1.0)*	4.6	0.007	0.16	T > S = P
*NDD students are prepared and trained for unstructured social situations*	0.7 *(1.1)*	1.4 *(1.2)*	1.6 *(1.2)*	3.2	0.023	0.11	T > S = P
**Staff education/professionalism (max. 6)**	2.8 *(1.6)*	2.8 *(1.5)*	3.8 *(1.3)*	1.9	0.069	0.10	
*The school staff has good knowledge of NDDs*	1.0 *(0.9)*	1.0 *(0.7)*	1.4 *(1.0)*	1.9	0.143	0.05	
*The staff understands that individualized support might be necessary for a student with NDD*	1.8 *(0.9)*	1.8 *(1.0)*	2.4 *(0.7)*	2.6	0.04	0.10	T > S = P

*Note*. Items have been translated from Swedish and shortened for the reader’s ease and summary presentation; ^a^ e.g., allowed to present orally instead of in written form, or vice versa; ^b^ e.g., can spend breaks in the classroom; ^c^ e.g., provide time-tables visualized schemes; ^d^ e.g., checklists, planning aids; *Neurodevelopmental disorder

## Results

See Table 1 for INCLUSIO item, domain, and the total scores across groups and complete inference statistics for group comparisons. The average student item score was 1.5. Student ratings were highest for the items, *There are regular exchanges between caregivers and responsible staff around students with NDD* (score 2.4, domain: *Cooperation with parents*) and *The school uses visualization of schedules and time* (score 2.2, domain: *Implementation of a structured learning environment*), and lowest for *NDD students are prepared and trained for unstructured social situations* (score 0.7, domain: *Consideration of peer-relations*) and *Changes to procedures are communicated to NDD students as early as possible* (score 0.9, domain: *Implementation of a structured learning environment*).

The average parent item score was 1.6. Parents scored the items *There is a specific and accessible support plan document for the students* (score 2.2) and *Staff involved in support plans meet regularly and support plans are evaluated* (both domain: *Assessment of support needs*) the highest. Low parent scores were found for *Strategies for handling stressful situations are provided* (score 0.8, domain: *Individual changes applied to schedule/teaching*) and *The school staff has good knowledge of NDD* (score 1.0, domain: *Staff education/professionalism*).

The average teacher item score was 2.0. Teachers rated the following items the highest: *Students are offered alternative options to demonstrate knowledge* (score 2.8, domain: *Use of individualized support*) and *In case of group work, the composition of the group takes into account knowledge of students with NDD* (score 2.5, domain: *Consideration of peer-relations*) and the following items the lowest: *Changes to procedures are communicated to NDD students as early as possible* (score 1.1) and *The school staff has good knowledge of NDD* (score 1.4).

**Table 2:** Informant Agreement (Kappas) for INCLUSIO Items

Domains / items	Kappa (*p*)
*Assessment of support needs*	
Recommendations from clinical services are used for support planning	− 0.21 (0.002)
There is a specific and accessible support plan document (IEP) and support plans are followed-up and evaluated	0.00 (0.99)
Staff involved in support plans meet regularly	− 0.19 (0.01)
*Use of individualized support*	
Students are offered alternative options to demonstrate knowledge ^a^	0.20 (0.03)
School rules are adapted to students’ needs ^b^	0.19 (0.01)
Students receive the individual special education support needed	0.01 (0.87)
Everyday individual adaptations in the classroom and schedule are provided	0.11 (0.21)
*Implementation of a structured learning environment*	
The school uses visualization of schedules and time ^c^	− 0.11 (0.16)
Students are offered organizational aids ^d^	0.04 (0.57)
Changes to procedures are communicated to NDD* students as early as possible	0.14 (0.03)
*Individual changes applied to teaching*	
Students’ interests are integrated in teaching	0.06 (0.38)
Strategies for handling stressful situations are provided	− 0.18 (0.02)
*Functional response to behavioral characteristics*	
The staff gets time to discuss NDD students’ behavior and support plans	0.05 (0.50)
The school offers space for rest and withdrawal	0.09 (0.26)
*Cooperation with parents*	
There is a mutual exchange of knowledge about students with NDDs between home and school	0.07 (0.39)
The school uses caregivers’ knowledge to optimize support	− 0.02 (0.82)
There are regular exchanges between caregivers and responsible staff around students with NDDs	− 0.09 (0.22)
*Consideration of peer-relations*	
In group work, the composition of the group takes into account the knowledge of students with NDDs	0.10 (0.16)
NDD students are prepared for unstructured social situations and trained in social interactions	0.11 (0.07)
*Staff education/professionalism*	
The school staff has good knowledge of NDDs	0.28 (0.001)
The staff understands that individualized support might be necessary for a student with NDD	0.03 (0.75)

*Note.* Items have been translated from Swedish and shortened for the reader’s ease and summary presentation; ^a^ e.g., allowed to present orally instead of in written form, or vice versa; ^b^ e.g., can spend breaks in the classroom; ^c^ e.g., provide time-tables, visualized schemes; ^d^ e.g., checklists, planning aids; *Neurodevelopmental disorder.

In line with our hypothesis, the total score of all INCLUSIO interview items was higher in teachers than in both students and parents (*p* = .001). The same was true for the domains *Use of individualized support* (*p* = .002) and *Functional response to behavioral characteristics* (*p* = .005), and 7 of the 21 items (*p* < .04). In addition, teacher scores were higher than student scores for the domain *Consideration of peer-relations* (*p* = .001) and higher than parent scores for the domain *Individual changes applied to schedule/teaching* (*p* = .01). Parents and teachers scored higher than students on the item *There is a specific and accessible support plan document, an individual educational plan (IEP) for the students* (*p* = .04), and students scored higher than teachers and parents for *The school uses visualization of schedules and time”* (*p* = .002). Students and teachers scored higher than parents on the item *Strategies for handling stressful situations are provided* (*p* = .001). Parents scored higher than students and teachers on the item *Changes to procedures are communicated to NDD students as early as possible* (*p* = .01).

Kappa values for agreement between groups for all INCLUSIO items are shown in Table 2. In total, 8 items yielded negative to no agreement (*r* = − .21 to .00), 12 yielded none to slight agreement (*r* = .01-.20), and 1 yielded fair agreement (r = .28, p = .001) [*The school staff has good knowledge of NDD*]. In the whole group, all INCLUSIO domains correlated highly (≥ *r* = .64) with the total score, and the domain *Use of individualized support* demonstrated the highest association (*r* = .85, *p* < .0001) with the overall inclusion score. All items correlated moderately to highly with the total score (*r* ≥ .33, p ≤ .006), and the items *The school uses the caregiver’s knowledge to optimize support* (*r* = .73, *p* ≤ .0001) and *The staff gets time to discuss NDD students’ behavior and support plans* (*r* = .72, *p* ≤ .0001) showed the closest association with the total score.

## Discussion

The comprehensive educational inclusion of autistic and other neurodivergent students is still far from being a reality in high- and middle-income countries, despite statements and legislation. A barrier to the implementation of educational inclusion is a lack of consensus about what inclusion should entail and the degree of inclusion accomplished. We investigated educational inclusion from the perspective of neurodivergent students, their caregivers, and teachers to generate awareness of possible disagreement as a basis to improve and guide better implementation practices. Consistent with previous findings (Bölte et al., 2021; Jones et al., 2022), the experience of implemented educational practice varied substantially between informants. There was disagreement between all informants, although mostly, but not exclusively, between students and parents versus teachers. Students and parents gave educational inclusion poor to adequate implementation ratings while teachers rated inclusion significantly higher, even though their evaluations suggested there was room for improvement. Students stated that they were not prepared for transitions and experienced limited transparency in case of changes during the school day. Transitions and limited predictability are known challenges for neurodivergent individuals (Mandy et al., 2016), and there are strategies to guide teachers in supporting students to master change (Borg et al., 2021). Students also experienced some practices as well-developed, such as visual supports in accordance with TEACCH principles (Virues-Ortega et al., 2013). Students and parents found that there was a culture of exchange with responsible school staff to discuss students’ situations. Parents agreed with teachers that there was adequate access to students’ support plans, but students did not share this view.

Educational legislation often states that all students have the right to be provided with a learning environment that enables them to reach their educational goals in a safe environment. Despite this, schools struggle to provide such environments for multiple reasons, such as due to a lack of best practice guidelines on inclusive education (Nilholm & Göransson, 2017; Nilholm, 2021) and limited knowledge about the needs of neurodivergent students (De Boer et al., 2011; Hume et al., 2021; Långh et al., 2017; Toye et al., 2019). Similarly, informants in our study agreed that the school staff’s knowledge was low, indicating that the education of school staff, especially teachers, is of paramount importance. However, in Sweden, for example, in teacher education at universities, only recently have a small number of higher education credits in NDDs been introduced as mandatory, and there are no specific requirements to focus on educational inclusion. Additionally, despite evidence that professional development with regard to inclusive action for neurodivergent students has beneficial effects (Petersson-Bloom & Bölte, 2022), this is not systematically provided.

Our findings imply that the most promising, essential and non-bureaucratic avenue to achieve consensus and progress for the educational inclusion of neurodivergent students in different schools and learning environments is the extended participation of students and parents in shaping practice and solutions. First, the domain *Use of individualized support* had the highest association with overall experience of inclusion, and students and parents are likely the best sources of information in this regard. Second, and corroborating the latter, the single measure that had the highest association with overall inclusion ratings was the school taking advantage of the caregiver’s knowledge to optimize support. Third, there were several indications from all informants that the schools had already established some culture of exchange between them, students, and parents. Enhancing the participation of neurodivergent students and their parents to achieve educational inclusion has been suggested by Florian (2014), Fletcher-Watson et al., (2019), and Lord et al., (2022) as a strategic research goal in the field.

Our results should be treated with caution because of methodological limitations inhibiting generalizability. A larger sample including a more diverse group of schools from other urban and rural areas would have been more desirable. We only investigated schools in Sweden, a high-income country with a strong social welfare infrastructure, although Sweden scores below the OECD average on the Programme for International Student Assessment. Data were collected during the COVID-19 pandemic, which might have affected the informants’ perceptions of educational inclusion. Other pivotal stakeholder groups, e.g., school leaders and policymakers of paramount importance for educational inclusion were omitted, but the study focused on a micro-environmental perspective, where informants should have access to comparable information.
